# Supplementary material for: Analysis of logistics capacity, influencing factors and spatial spillover effect in Yangtze River Economic Belt
Source: PLoS One. 2024 May 22;19(5):e0303200. doi: 10.1371/journal.pone.0303200 (PMC11111048; doi:10.1371/journal.pone.0303200)
Supplement: Attachment — Submitted filename: Response to Reviewers.docx [file pone.0303200.s002.docx]

**Response to Reviewer Comments**

Dear Reviewer,

It is our great honor to receive the comments on my paper from you. Those comments are all valuable and very helpful for revising and improving our paper.

We have studied the comments carefully and have made corrections which we hope meet the approval. Revised portions are marked in blue on the paper. You can find them in the attached files uploading. If there are any errors, please notify us. We will correct them accordingly.

Once again, thank you very much for your comments and suggestions.

**Comment 1**: *The authors should ensure that the revised manuscript is free of punctuation errors and hat the references are formatted consistently with the journal’s guidelines.*

**Response 1:** We thank the reviewer for raising this question. We should do the work that the sentences of articles are smooth, the punctuation is standardized and the format of articles conforms to the requirements of periodical format. We will carefully revise to ensure that such issues are addressed. Thank you very much!

**Revise 1:**(pages 26-28 lines 853-956)

People's Republic of China. Guiding Opinions of The State Council on Promoting the development of the Yangtze River Economic Belt by relying on the Golden Waterway. 2014,9. Available from: https://www.gov.cn/gongbao/content/2014/content_2758494.htm

People's Republic of China. The National Development and Reform Commission issued a notice on the Layout Plan of the Yangtze River Trunk Crossing Passage (2020-2035). 2020,3. Available from: https://www.gov.cn/zhengce/zhengceku/2020-04/08/content_5500124.htm

Zawawi NFBM, Wahab SA, Mamun AA, et al. Logistics Capability, Information Technology, and Innovation Capability of Logistics Service Providers: Empirical Evidence from East Coast Malaysia. International Review of Management & Marketing, 2017, 7.

Wei HL, Jian HJI. Research on the management process of logistics capabilities in supply Chain. Journal of Chongqing Jiaotong University, 2006.

Bowersox DJ, Closs DJ. Logistical management: the integrated supply chain process(vol.2). 1999.

Daugherty, Patricia J, Pittman, et al. Utilization of time-based strategies: Creating distribution flexibility/responsiveness. International Journal of Operations & Production Management, 1995. doi:10.1108/01443579510080418.

Fawcett SE, Stanley LL, Smith SR. Developing a logistics capability to improve the performance of international operations. Journal of Business Logistics, 1997; 18(2):101-127.

Lynch, Daniel F, Scott B. Keller, John O. The effects of logistics capabilities and strategy on firm performance. Journal of business logistics. 2000; 21(2): 47.

Zhao M, Theodore PS. Interactions between operational and relational capabilities in fast food service delivery. Transportation Research Part E: Logistics and Transportation Review .2003; 39(2): 161-173.

Bharadwaj S, Bharadwaj A, Bendoly E. The Performance Effects of Complementarities Between Information Systems, Marketing, Manufacturing, and Supply Chain Processes. Information Systems Research, 2007; 18(4):437-453. doi:10.1287/isre.1070.0148.

Wu F, Yeniyurt S, Kim D, et al. The impact of information technology on supply chain capabilities and firm performance: A resource-based view. Industrial Marketing Management, 2006; 35(4): 493-504. doi:10.1016/j.indmarman.2005.05.003.

Li JJ. Competitive position, managerial ties, and profitability of foreign firms in China: an interactive perspective. Strategic Direction, 2009; 25(8). doi:10.1108/sd.2009.05625had.008.

Lynch D, Keller S, Ozment J. The effects of logistics capabilities and strategy on firm performance. Journal of business logistics. 2000; 21 (2): 47.

Ackermann J, Egon M. Modelling, planning and designing of logistics structures of regional competence-cell-based networks with structure types. Robotics and Computer Integrated Manufacturing, 2007; 23(6):601-607. doi:10.1016/j.rcim.2007.02.010.

Lu H. A model of integrated regional logistics hub in supply chain. Enterprise Information Systems. 2018; 12(10): 1308-1335.

Guo HP. MLP neural network-based regional logistics demand prediction. Neural Computing and Applications.2021; 33 (9): 3939-3952.

Heng C, Shan YJ, Zeng T. An Analysis of the Development Mode and Influence Effect of Regional Logistics Industry in China. Commercial Research. 2020; 62(5): 55.

18Zhang D, Eglese R, Li S. Optimal location and size of logistics parks in a regional logistics network with economies of scale and CO2 emission taxes. Transport, 2015; 33(1):1-17. doi:10.3846/16484142.2015.1004644.

Zhao W, Zhang J, Zhao L. Research on Evaluation of Regional Logistics Capability under Big Data Background. MATEC Web of Conferences, 2017; 100.

Carlucci F ,Cirà, Andrea, Forte E . Infrastructure and logistics divide: regional comparisons between North Eastern & Southern Italy. Technological & Economic Development of Economy. 2017;1-27.

Huang X, Wang Y, Dai X, et al. Evaluation of port efficiency in shanghai port and busan port based on three-stage DEA model with environmental concerns. Transport . 2019; 35(5):1-8. doi:10.3846/transport.2019.11465.

Hylton PJ, Ross CL. Agglomeration economies' influence on logistics clusters' growth and competitiveness. Regional Studies. 2018;1-12.

Sun T, Nong P, Weng SZ. Comprehensive evaluation of regional logistics development level in Guangxi based on fuzzy matter-element analysis . Logistics Engineering and Management. 2019; 44(06):6-9+13.

Jiang H, Sun TP, Zhuang BN. Determinants of Low-Carbon Logistics Capability Based on Dynamic fsQCA: Evidence from China's Provincial Panel Data. Sustainability. 2023; 15(14):7-12.

Zhang GS, Xiang WM. Research on evaluation of regional logistics capacity of four major urban agglomerations in China. Journal of Chongqing Technology and Business University (Social Science Edition). 1-13 http://kns.cnki.net/kcms/detail/50.1154.c.20231109.1726.006.html.

He XG, Lu GD. The influence of regional logistics capability on the performance of circulation enterprises: Based on empirical evidence of listed companies in circulation industry. Business Economics Research, 2023; 6(21): 26-29.

Shi PZ. Research on evaluation and improvement path of regional logistics capability under new development Concept: A case study of Henan Province. Journal of Henan Polytechnic University (social sciences Edition).2023; 24(04): 31-41.

Pavić, Zlatko, Vedran Novoselac. Notes on TOPSIS method. Int. J. Res. Eng. Sci. 2013;1 (2): 5-12.

Shi T, Si SC, Chan J. The Carbon Emission Reduction Effect of Technological Innovation on the Transportation Industry and Its Spatial Heterogeneity: Evidence from China. Atmosphere. 2021;12(9):23-26.

Aleksy K, Oleksii L, Tetyana P. Spillover Effects of Green Finance on Attaining Sustainable Development: Spatial Durbin Model. Computation. 2023; 11(10): 12-21.

Zhang C. Research on the Economical Influence of the Difference of Regional Logistics Developing Level in China. Journal of Industrial Integration and Management, 2020. doi:10.1142/S2424862220500049.

Guo ZX, Tian Y, Guo XM, et al. Research on Measurement and Application of China's Regional Logistics Development Level under Low Carbon Environment. PROCESSES, 2021,9(12).

Yu N, Xu W, Yu KL. Research on Regional Logistics Demand Forecast Based on Improved Support Vector Machine: A Case Study of Qingdao City under the New Free Trade Zone Strategy. IEEE ACCESS, 2020,8:9551-9564.

Liu J. Development of Regional Logistics Along the One Belt and One Road. Springer Singapore, 2016; 77-101.

China Statistical Yearbook. 2016-2020. Available online: http://www.stats.gov.cn/sj/ndsj/

LeSage J, Pace RK. Introduction to Spatial Econometrics. New York: Chapman and Hall/CRC, 2009.

**Comment 2**: *The authors should provide a more detailed explanation of the methodology used, especially the rationale behind the selection of the spatial weighting matrix and the interpretation of the spatial econometric model results.*

**Response2:** Thank you for your very good advice. We have added a description of the methodology used in the correspondence section.

**Revise 2:**(pages 8,9 lines303-320)

The traditional measurement model only studies the influence of individual independent variables on individual dependent variables, while the spatial measurement model further explores the influence of individual independent variables on other individual dependent variables on the basis of traditional measurement. Therefore, the greatest feature of spatial metrology is to fully consider the spatial correlation between cross-sectional units. Spatial correlation refers to the spatial interdependence, mutual restriction, mutual influence and interaction between things and phenomena in different regions. It is the inherent spatial economic attribute of things and phenomena, and the essential attribute of geographical spatial phenomena and spatial processes. The spatial correlation is caused by the proximity of geographical location, the competition and cooperation of individuals at the cross-section level, the group imitation behavior, the spillover effect of economic activities and the measurement error. Therefore, in the actual construction of the model, how to incorporate the spatial effects of economic variables into the model, it is necessary to establish a spatial weight matrix according to some standards. To define the spatial weight, we must first quantify the position of the spatial unit, which usually includes neighborhood space distance, geographical distance, economic distance and industrial distance.

**Comment 3**: *The authors should consider discussing the limitations of the study, such as the potential for spatial heterogeneity within provinces and the assumptions made in the spatial econometric model.*

**Response 3:** Thank you for your suggestion. This is a very good suggestion. According to your suggestion, we have added content to the article about the limitations of the study.

**Revise 3:**(page25,26 lines 836-851)

It should be noted that this study also has some limitations. First, it is limited by the availability of data. This paper only uses the panel data of provinces and cities in the Yangtze River Economic Belt from 2016 to 2020 for empirical research. Future research may consider using the data of prefecture-level cities to get more specific conclusions and put forward more specific policy recommendations. Second, there is no unified standard for the evaluation of logistics capability. Although 20 indicators were selected to measure the level of logistics capacity in various provinces and cities, it is difficult to fully consider the influencing factors of logistics capacity in the research due to the extensive content of logistics capacity and the difficulty in obtaining some measurement indicators. Therefore, how to accurately measure the regional logistics capability is still a problem that needs to be solved in the future. Finally, in the analysis of the spatial spillover effect of the regional logistics capacity of the Yangtze River Economic Belt, only some factors are selected for spatial factor analysis, which may have the influence of spatial autocorrelation of unobserved factors, resulting in missing variables bias. Relevant influencing factors can be further enriched in the subsequent research.
